# Ecosystem engineering and leaf quality together affect arthropod community structure and diversity on white oak (*Quercus alba* L.)

**DOI:** 10.1007/s00442-023-05439-1

**Published:** 2023-09-09

**Authors:** Jason R. Reinhardt, Robert J. Marquis

**Affiliations:** 1 Department of Biology and the Whitney R. Harris World Ecology Center, University of Missouri-St. Louis, 1 University Boulevard, St. Louis, MO 63121 USA; 2 USDA Forest Service, Rocky Mountain Research Station, Forest and Woodland Ecosystems, 1221 South Main Street, Moscow, ID 83843 USA

**Keywords:** Community assembly, Ecosystem engineering, Habitat diversity, Leaf phenolics, Interspecific interactions, Leaf tying caterpillars, Lepidoptera, Stress-gradient hypothesis, Habitat diversity hypothesis

## Abstract

Shelter building caterpillars act as ecosystem engineers by creating and maintaining leaf shelters, which are then colonized by other arthropods. Foliage quality has been shown to influence initial colonization by shelter-building caterpillars. However, the effects of plant quality on the interactions between ecosystem engineers and their communities have yet to be studied at the whole plant level. We examined how leaf tying caterpillars, as ecosystem engineers, impact arthropod communities on *Quercus alba* (white oak), and the modifying effect of foliage quality on these interactions. We removed all leaf tying caterpillars and leaf ties on 35 *Q. alba* saplings during the season when leaf tying caterpillars were active (June–September), and compared these leaf tie removal trees to 35 control trees whose leaf ties were left intact. Removal of these ecosystem engineers had no impact on overall arthropod species richness, but reduced species diversity, and overall arthropod abundance and that of most guilds, and changed the structure of the arthropod community as the season progressed. There was an increase in plant-level species richness with increasing number of leaf ties, consistent with Habitat Diversity Hypothesis. In turn, total arthropod density, and that of both leaf tying caterpillars and free-feeding caterpillars were affected by foliar tannin and nitrogen concentrations, and leaf water content. The engineering effect was greatest on low quality plants, consistent with the Stress-Gradient Hypothesis. Our results demonstrate that interactions between ecosystem engineering and plant quality together determine community structure of arthropods on *Q. alba* in Missouri.

**Supplementary Information:**

The online version contains supplementary material available at 10.1007/s00442-023-05439-1.

## Introduction

Numerous herbivorous insect species modify plant structure via shelter building (Lill and Marquis 2006). The tents, leaf rolls, leaf ties, frass chains, silk webs, stem bores, and leaf mines made by these shelter builders are often colonized by other arthropods, including herbivores, detritivores, predators, and parasitoids (e.g., Fukui 2001; Vieira and Romero 2013; Novais et al. 2020; Marquis et al. 2022). These colonizers can come from other parts of the same plant or newly colonize the plant (Lill and Marquis 2004). In the former pathway, the arthropod fauna is re-organized with no change in composition, while in the latter the faunal structure may be modified greatly at the whole plant level (e.g., Lill and Marquis 2003; Vieira and Romero 2013; Wetzel et al. 2016). In both cases, these shelter builders act as ecosystem engineers: they modify the physical structure of the environment, which in turn influences resource availability for associated species (Jones et al. 1997).

Impacts of shelter building on arthropod community structure at the whole plant level are widespread: the engineers that produce these whole plant effects are diverse (leaf tying, leaf rolling, and stem boring caterpillars, and gall making insects), they impact faunas on plants of diverse growth forms (trees, shrubs, herbaceous perennials, and vines), and they do so in both tropical and temperate regions (Lill and Marquis 2003; Crawford et al. 2007; Vieira and Romero 2013; Harvey et al. 2016; Henriques et al. 2019; Wetzel et al. 2016; Pereira et al. 2021).

Although important for understanding the factors that structure arthropod faunas on plants (Strong et al. 1984), there has been little study of the contribution of plant traits to the impact of shelter building insects as ecosystem engineers. Host plant traits likely contribute to the relevant processes in various ways. First, plant quality, in the form of foliar nutrients and defensive chemistry, may influence the initial level of colonization by the shelter building species. There are many examples of leaf quality influencing the abundance of leaf tying caterpillars (e.g., Marquis and Lill 2010), leaf rolling caterpillars (e.g., Singh et al. 2022), galls (e.g., Helms et al. 2013), leaf mines (e.g., Yarnes et al. 2008), leaf tents (Aide and Londono 1989), stem boring (Joo et al. 2021), and webs (Wang et al. 2017) on their host plants. In turn, plant architecture influences attack by both leaf tying caterpillars (Marquis et al. 2002) and leaf rolling beetles (Higuchi and Kawakita 2019). Second, plant traits may influence the abundance and identity of non-shelter building species prior to initial shelter building, thus determining the local pool of potential colonists of newly made shelters (Wang et al. 2012). Third, plant traits can modify the attraction of colonists from off the plant, after it has been colonized by shelter builders (Lill and Marquis 2004). Finally, shelter building may modify the quality of leaves in shelters, via local induction, and in so doing, influence their use by the herbivore component of colonizers (Sagers 1992; Fukui et al. 2002; Wang et al. 2023).

Relevant studies measuring the impacts of plant traits on arthropod community structure at the whole plant level via shelter building have not been conducted. Here, we report the results of an experiment designed to test the role of leaf quality in modifying the impact of leaf tying caterpillars on the arthropod fauna of their host plant, *Quercus alba* L. (Fagaceae, white oak). Oaks in the eastern United States and adjacent Canada host a number of shelter-building caterpillar species (Marquis et al. 2019b). One type of shelter is a leaf tie, produced when caterpillars tie two or more overlapping leaves together with silk (Carroll 1977; Carroll and Kearby 1978; Carroll et al. 1979). Once formed, the shelter builder feeds within the tie, which accumulates frass, silk, undigested leaf particles, and insect body parts as it ages. The shelter may also provide protection against the abiotic environment and natural enemies (Fukui et al. 2002; Marquis et al. 2022). As a result, the shelter and its contents serve as a resource for other species that then colonize or visit the leaf tie.

Previous studies of leaf tying caterpillars on *Quercus alba* have shown that leaf ties are significant colonization sites for non-leaf tying arthropod species that otherwise might not colonize the plant or do so in low abundance (Lill and Marquis 2004; Wang et al. 2012; Baer and Marquis 2014). Removal of leaf tying caterpillars for a three-week period in early summer had significant effects on insect herbivore diversity and abundance (Lill and Marquis 2003, 2004). Additional studies have shown that leaf quality variation within and among oak species can influence attack by insect herbivores and leaf tying caterpillars (Forkner et al. 2004; Lill and Marquis 2001; Marquis and Lill 2010). No studies have yet examined the season-long arthropod community level impacts of leaf tying caterpillars in conjunction with leaf quality. In addition, no studies have assessed the influence of plant traits, either architecture (Marquis et al. 2002; Marquis and Lill 2010) or leaf quality, on ecosystem engineering impacts at the whole plant level.

Here, we ask how leaf quality modifies the impact of ecosystem engineers on the associated arthropod fauna on their host plants. Specifically, we hypothesized that (1) ecosystem engineering by leaf tying caterpillars would affect the structure of the non-leaf tying arthropod community; and (2) that leaf tying and subsequent colonization of the ties would be influenced by leaf quality, with the prediction that higher leaf quality would lead to greater colonization by leaf tying caterpillars and increased abundance of arthropods on experimental trees. In addition, we tested two hypotheses derived from ecosystem engineering theory and applied to our system. The first, the Habitat Diversity Hypothesis (HDH), predicts that plants with intermediate leaf quality will have intermediate numbers of leaves modified as shelters (Williams 1964; Hastings et al. 2007). These plants are predicted to have the highest arthropod richness because non-tied leaves and leaves in shelters will be relatively equal in abundance thus supporting both arthropod species that specialize on non-tied leaves and those that depend on leaf shelters (Marquis and Lill 2007). In contrast, when leaf tie abundance is low or zero, species that require leaf ties will be absent or low in abundance; when leaf tie abundance is very high, species that avoid leaf ties will be absent or low in abundance. The Stress-Gradient Hypothesis (SGH) (Crain and Bertness 2006; He and Bertness 2014), in contrast, posits that the greatest positive effect of ecosystem engineering on arthropod abundance and species composition will occur in the most stressful environments. For arthropods on plants, plant defenses in particular and plant quality in general represent one important aspect of environmental stress (Berenbaum 1995; Johnson and Felton 2001; Awmack and Leather 2002; Huberty and Denno 2004; Forkner et al. 2004; Johnson 2011). Accordingly, we predicted that the impact of leaf shelters on components of arthropod community structure would be greatest on plants with poor leaf quality. As far as we are aware, there has been no test of the Habitat Diversity Hypothesis and only one test of the Stress-Gradient Hypothesis (Vieira and Romero 2013) as they apply to insect herbivores acting as ecosystem engineers.

## Methods

### Study site and system

Field experiments were conducted at Cuivre River State Park (CRSP), located in Lincoln County, Missouri, USA (39°02′01″N 90°55′51″W/39.03361°N 90.93083°W, average elev. 567 m). The park is approximately 25.9 km^2^ and consists mainly of forest, savanna, and managed prairie. The park contains a second-growth mixed oak-hickory forest with an understory composed largely of flowering dogwood (*Cornus florida*), sassafras (*Sassafras albidum*), sugar maple (*Acer saccharum*), eastern redbud (*Cercis canadensis*), and an assortment of saplings, including many oaks (*Quercus* spp.). The canopy is dominated by oak (*Quercus* spp.) and hickory (*Carya* spp.).

*Quercus alba*, the focal plant of this study, is one of the most common species of oak in both canopy and understory of CRSP. Most leaf tying caterpillar species that attack *Q. alba* have two generations, one that emerges in early to mid-June and one beginning in mid- to late August (Lill 2004; Marquis et al. 2019b). Leaf tying caterpillars present in Missouri oak communities represent several families, including Gelechiidae, Depressariidae, Hesperiidae, Noctuidae, Pyralidae, and Tortricidae (Lill and Marquis 2003; Forkner et al. 2004; Marquis et al. 2019b). The most common leaf tying caterpillar species found on *Q. alba* in Missouri are *Psilocorsis quercicella, P. cryptolechiella*, and *P. reflexa* of the Depressariidae, and *Arogalea cristifasciella* and *Pseudotelphusa quercinigracella* of the Gelechiidae (Lill and Marquis 2003; Forkner et al. 2004). Of these, *P. quercinigracella* is often the most abundant species, and has a large impact on the insect herbivore community (Lill and Marquis 2003). The most abundant arthropods on Missouri oaks in general, other than leaf tying caterpillars are Lepidoptera, Homoptera, Thysanoptera and Psocoptera (Lill and Marquis 2004). Coleoptera and Araneae are also fairly abundant, but to a lesser extent.

### Experimental design

In early June 2009, seventy white oak saplings at the study site were marked and their leaves counted. Trees were alternately assigned to either treatment (leaf ties removed) or control (leaf ties intact). Trees were 1–4 m tall, with leaf counts ranging between 300 and 900. From mid-June to mid-September, all leaf tying caterpillars were removed from all treatment trees 7–30 days (Table S1). Any leaf ties constructed were recorded and disassembled. The identity and quantity of all leaf tying caterpillars removed were recorded (Table S1). Only leaf tying caterpillars were removed. As a procedural control, the control tree in each pair was visited and handled at the same time as the treatment tree. A mean of 7.1 (± 1.2 SE) caterpillars were removed per plant (range 1–21) over the course of the experiment (Table S1).

We conducted censuses in late June/early July, August, and September for the abundance and identity of arthropods. Each leaf on each tree was carefully searched and arthropod species were counted and identified to morphospecies. Caterpillars were collected only if they were unidentifiable to morphospecies in the field; in that case, they were taken to the laboratory for identification, and reared to maturity. Small metal clips (Sally Beauty Supply) were used to keep leaf ties intact that were opened on control trees. These clips were removed on the next visit, 4–7 days later.

Leaf quality data were collected from all study trees: leaf toughness, water content, percent dry weight carbon and nitrogen, and concentration of condensed tannins, hydrolysable tannins, and total phenolics. Five mostly undamaged leaves (< 5% leaf area missing) from each sapling were collected for analysis at the beginning (June/July) and end of the experiment (September) to determine how leaf quality changed over the course of the season. Collected leaves were kept on ice, returned to the laboratory and freeze-dried, ground into powder, and stored in a − 80 ºC freezer. Leaf toughness was measured at the time of collection using a leaf penetrometer (Force Dial FDK 32, Wagner Instruments, Greenwich, CT). Water content was measured by subtracting the dry weight from the weight at collection. Percent dry weight carbon and nitrogen were measured using a CHNS/O analyzer, which uses microcombustion to break down and estimate the elemental composition of organic samples (Perkin-Elmer Series II CHNS/O Analyzer 2400). Condensed tannin concentrations were determined using the acid-butanol technique (Rossiter et al. 1988). Hydrolysable tannin concentrations were estimated using the potassium-iodate assay (Schultz and Baldwin 1982). Total phenolic concentrations were estimated using the Folin-Denis assay (Waterman and Mole 1994). For the phenolic assays, a single bulk standard containing leaf tissue from each tree was prepared and purified by washing the leaf powder multiple times with 95% ethanol, followed by extraction using 70% acetone with Sephadex LH-20 in a Büchner funnel. Acetone was removed using rotary evaporation, leaving pure oak tannin in an aqueous solution. The aqueous solution was freeze-dried, leaving only purified oak tannin powder. Individual aqueous extracted samples were obtained by purifying with multiple 95% ethanol washes followed by extraction with 70% acetone. Individual samples were compared with the bulk standard for each assay, and colorimetrically quantified using a microplate reader (VersaMax Microplate Reader, Molecular Devices Corporation, Sunnyvale, CA).

### Statistical analyses

All statistical analyses and graphical presentations were completed using R version 4.1.0 (R Core Team 2021).

### Hypothesis 1: leaf ties affect arthropod community structure

To determine the effects of leaf tie removal on total arthropod species richness (Hill number q = 0), Shannon diversity (q = 1), and inverse Simpson diversity (q = 2), individual-based rarefaction curves, with extrapolations to twice as many as the lowest number of individuals as actually sampled, were constructed for both treatments for each of the three censuses using iNEXT (Chao et al. 2016). Leaf tying caterpillars removed from trees of the removal treatment were not included in the analyses. Treatments were considered to be significantly different in q values when the 95% confidence intervals of the two different treatments did not overlap (Colwell et al. 2012).

To assess how the treatments affected overall arthropod abundance, we first categorized each observed species or morphospecies into one of nine guilds (Table S2). We then compared the overall density (number of arthropods per leaf) and density of each guild during each census using repeated measures ANOVA, with treatment as a between-subjects factor. We tested for a season effect on the density of leaf ties and leaf tying caterpillars using a randomized block ANOVA with tree as the block and season as the treatment, including control trees only.

We used nonmetric multidimensional scaling (NMDS), permutational multivariate analysis (perMANOVA), and random forest classification analysis (RF) to explore compositional differences in the arthropod community between the control and removal treatments. Bray Curtis dissimilarity indices (Bray and Curtis 1957) were used in the construction of the NMDS, and the perMANOVA allowed us to determine whether treatments differed significantly in terms of community composition. A RF classification analysis (Breiman 2001) was used to determine if there were differences in guild-based arthropod community structure between treatments, and if so, which guilds contributed most to the differences. NMDS does not allow either. RF is a decision tree-based, machine learning tool which can be used for either classification or regression analyses (Breiman 2001; Cutler et al. 2007). It is an ensemble modeling approach in which many individual regression or classification trees are constructed, each using a bootstrapped sample from the full dataset. Each tree contains only a subset of all available predictor variables, and predictor variables are recursively partitioned within each tree (Breiman 2017). Because each tree is trained using a bootstrapped sample of the full dataset, each tree therefore has a set of ‘out-of-bag’ data that are available for predictive performance analysis. The aggregation of these trees (i.e., the ensemble) is used as the full RF model (Breiman 2001, 2017). The relative importance of each variable in the RF analysis is assessed by shuffling out-of-bag data from each variable in the analysis and assessing how classification accuracy changes as a result (Breiman 2001); additional variable importance is assessed by examining the impact of each variable on group homogeneity (Gini) within each tree (Breiman 2001). All RFs were constructed using 500 trees and implemented using the *randomForest* package in R (Liaw and Wiener 2002).

### Hypothesis 2: leaf quality influences leaf tie formation and arthropod community composition

Repeated measures ANOVA, with treatment as the independent variable, was used to determine seasonal differences in all of the measured leaf quality traits: water content, toughness, concentrations of condensed tannins, hydrolysable tannins, and total phenolics, and percent dry weight carbon and nitrogen. To normalize the residuals, toughness was log_10_ (*x* + 1) transformed, and water content, condensed tannins, hydrolysable tannins, total phenolics, and percent dry weight carbon and nitrogen were logit transformed.

To assess the effects of leaf quality on the arthropod community, the number of leaf quality variables was reduced using principal components analysis (PCA). The transformed leaf quality data (described above) were used to maintain linear relationships. Total phenolic concentrations were omitted from the analysis due to a high correlation with hydrolysable tannins (r > 0.5;* P* > 0.001). Toughness was omitted from the analysis due to a high correlation with water content (r > 0.5; *P* > 0.05). Carbon and nitrogen were included as the C:N ratio. A ratio representing the nitrogen to phenolics relationship was included as N:hydrolysable tannins. Correlations among the various leaf quality factors are provided in Table S3. Separate PCAs were performed, one for July and one for September. Analyses were performed using the correlation matrix.

To determine the effects of leaf quality on leaf tying caterpillar colonization throughout the season, leaf quality principal components (PCs) were (Pearson) correlated with the number of leaf ties and leaf tying caterpillar species per leaf on control trees in July and September. Leaf quality effects on density of all arthropods and that of individual guilds were assessed using Pearson correlations with PC values from June/July and September.

### Hypothesis 3: habitat diversity hypothesis

Both species density (number of species per leaf including leaf tying caterpillars) and density of arthropods (numbers per leaf) were regressed on the proportion of leaves tied on control plants. Hypothesis 3 predicts that both would increase with the number of leaf ties, but would decline at high levels of leaf tying caterpillar attack. We calculated whether a curvilinear relationship exists for either relationship by testing whether a significant additional amount of sum of squares was explained when adding a quadratic term to the two regression equations (Zar 1999).

### Hypothesis 4: stress-gradient hypothesis

To test this hypothesis at least two kinds of habitats are needed, low stress and high stress. We partitioned control and treatment trees into each of two groups based on leaf characteristics, the low quality group having high concentrations of condensed and hydrolysable tannins, high toughness, and low nitrogen (high stress), with the high quality group the opposite for all four variables (low stress). Categories were created by comparing the variable loadings of leaf quality PCs for both July and September, ranking them based on their association with high nitrogen and low tannin concentrations. Groups were formed by splitting plants at the 50^th^ percentile rank for both control and treatment, yielding equal sample sizes (Table S4). Separate two-way repeated measures ANOVAs were used to determine how treatment effects on arthropod species richness, diversity, and abundance differed between quality levels over the season. Treatment and leaf quality group were used as between-subjects factors. We tested the SGH by specifically looking for a significant interaction between treatment and leaf quality (either high or low). The SGH predicts a greater treatment impact on response variables (diversity, richness, or density) for low quality plants than for high quality plants.

## Results

### Hypothesis 1: leaf ties affect arthropod community structure

A total of 11,696 individuals from 105 morphospecies of arthropods were recorded across all trees and treatments (Table S2). The second generation of leaf tying caterpillars appeared in mid- to late August, just after the second census. The mean density of leaf ties found on control trees ranged from 0.019 ties/leaf in July to 0.009 ties/leaf in August and 0.013 in September (Fig. S1a). Leaf tying caterpillars represented on average 13.20%, 2.06%, and 12.75% of the total arthropods found on control plants for the July, August, and September censuses, respectively (Fig. S1b). Despite these relatively low numbers of leaf ties, compared to past studies (e.g., Lill and Marquis 2003) and long term trends (Marquis et al. 2019a), the experimental removal of ties influenced the diversity, abundance, and community structure of the arthropods at the whole plant level.

### Arthropod diversity and abundance

**Fig. 1:**
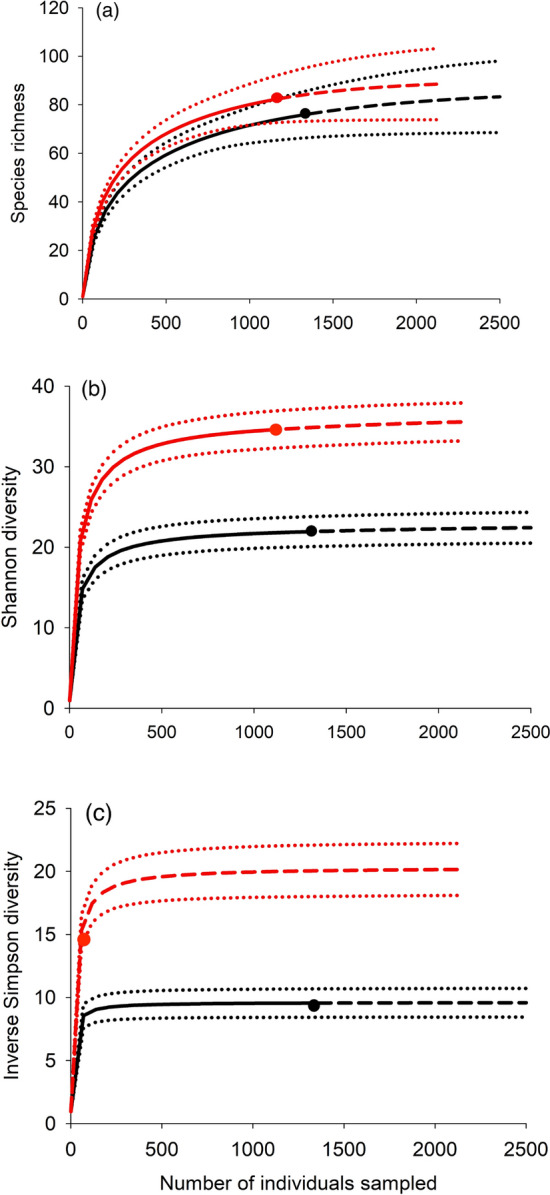
Rarefaction and extrapolation curves for all arthropods excluding leaf tying caterpillars censused in September 2009 for: a) species richness (Hill number q = 0), b) Shannon diversity (q = 1), and c) the inverse of Simpson diversity (q = 2), based on iNEXT (Chao et al. 2016). Red = control treatment, black = removal treatment. Dark solid lines are the rarefaction curves, and connected dashed lines are extrapolations. Dotted lines are the 95% confidence intervals. Large filled dots indicate the observed values for the associated treatment and metric

Treatment effects on plant-level species richness and diversity were modest, and greatest at the end of the experiment. In September, control trees had 50% higher Shannon diversity (q = 1) and 100% higher reciprocal Simpson diversity (q = 2) than removal trees but not higher species richness (q = 0) (Fig. 1). There were no significant differences between control and removal trees in any of the three measures of diversity for the June-July and August censuses.

**Fig. 2:**
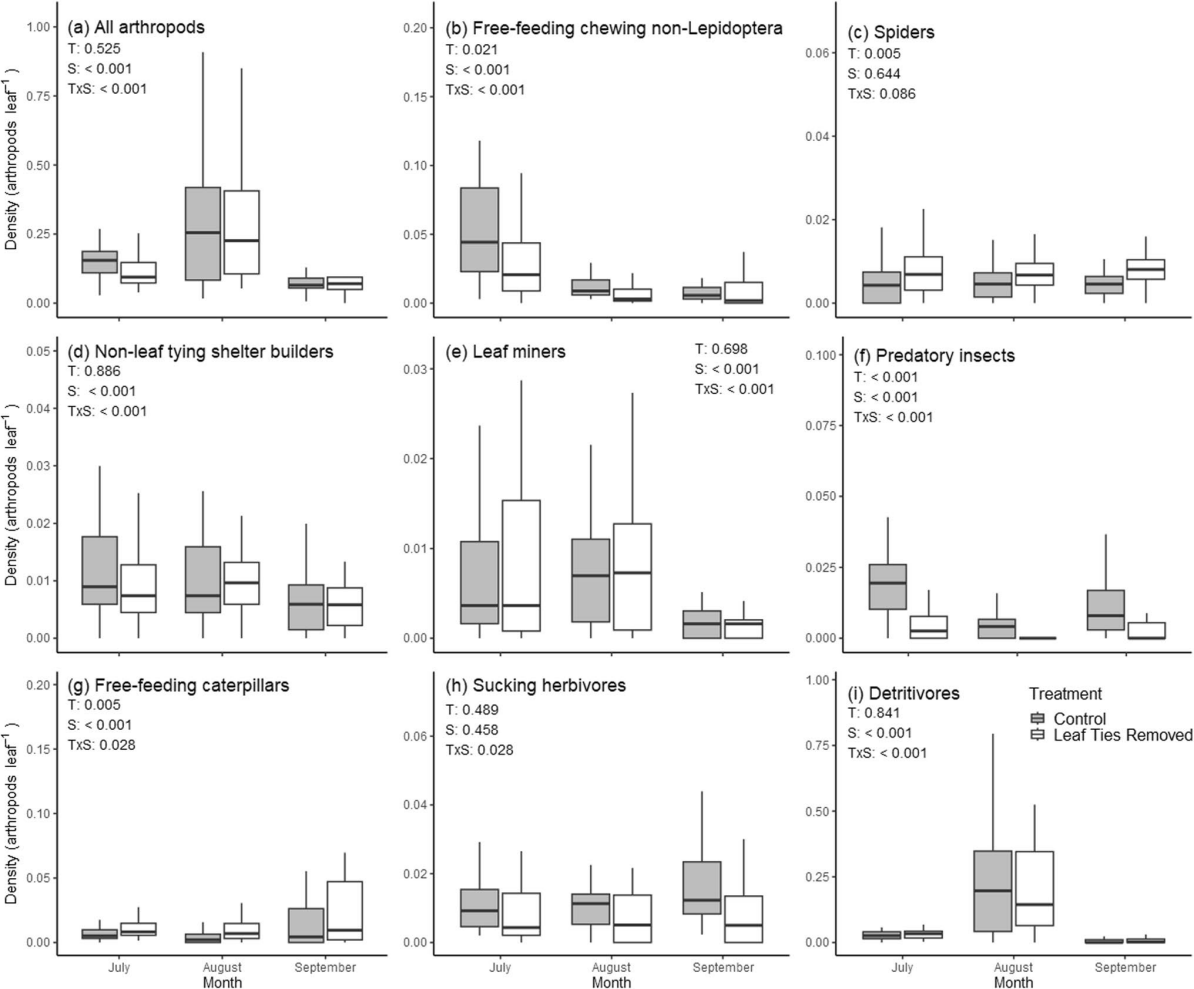
Treatment (control vs. leaf tie removal) and census effects on the density of all arthropods and of individual guilds. These are boxplots, showing medians (horizontal line) within the box. The upper edge of the box represents 75% of the data and lower edge 25% of the data, with the two horizontal lines containing 95% of the data. *P* values for treatment, census, and treatment × census effects are given in the upper left hand corner for each graph (see also Table S4)

In contrast to the effects on species richness and diversity, treatment affected the density of a large portion of the arthropod fauna, with the treatment effect dependent on season (strong treatment × season interactions) for all guilds but spiders (Fig. 2, Table S5). Treatment effects took place against a background of strong seasonal changes in the fauna, as seven of nine guilds changed in density seasonally, as did total arthropod density (season effect: Table S5). The treatment increased the total number of arthropods in the first census but not in the latter two (significant treatment × census interaction) (Fig. 2). Even stronger effects of leaf tie removal were seen for individual guilds. Treatment affected the density of five of eight guilds of arthropods and was dependent on season for seven of eight guilds including total arthropod density. Non-leaf tying shelter builders, predatory insects, sucking herbivores, and detritivores all decreased with leaf tie removal, while leaf miners, spiders, and free-feeding caterpillar species increased when ties were removed. Treatment × season interactions were particularly strong for free-feeding non-lepidopteran chewing herbivores (Coleoptera and sawflies) and sucking herbivores: treatment effects on the former were greatest in August (almost reduced by half), and doubled for the latter in September (Fig. 2).

### Community composition and structure

**Fig. 3:**
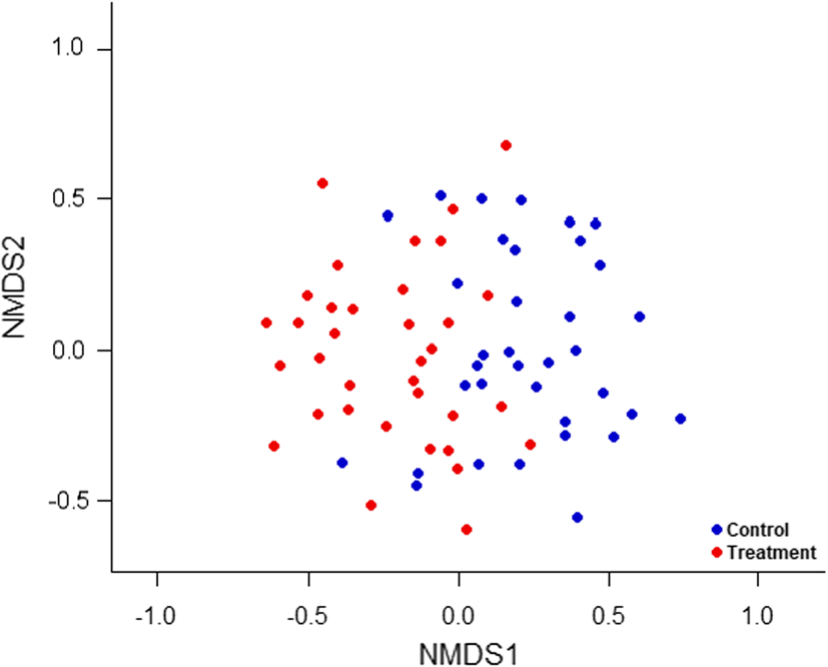
Effect of treatment (control vs. leaf tie removal) on arthropod composition, using nonmetric multidimensional scaling of arthropod community data across all three censuses. Blue dots represent control trees, red dots represent treatment trees

All NMDS ordinations were successfully constructed using two dimensions (2D stress values: full = 0.10, June = 0.14, August = 0.07, September = 0.15) (Fig. 3, Fig. S2). Each ordination also met the perMANOVA assumption of multivariate homogeneity of group variances (β-dispersion test: full *P* = 0.885, June *P* = 0.331, August *P* = 0.710, September *P* = 0.506). We found compositional differences in the arthropod community between the control and removal treatments in the full (aggregated) dataset (*P* = 0.013), in June (*P* = 0.001), and in September (*P* = 0.008), but not in August (*P* = 0.241). These compositional differences demonstrate that leaf tying caterpillars altered plant-level community composition, and that the impacts were least apparent mid-season when leaf tie abundance was lowest (i.e., August: between caterpillar generations).

Random forest classification analysis suggested that guild-based arthropod community structure differed between treatments, and that the differences increased over time. In the full (aggregate) dataset, the out-of-bag classification error rate was 35.71%; this reflects the generally decreasing monthly error rates of June-July = 44.29%, August = 37.14%, and September = 25.14%. In the full dataset, free-feeding caterpillars and non-lepidopteran chewing herbivores were the most important guilds in terms of classification accuracy, followed by spiders and predatory insects (Table 1). June and September results were largely similar to the full dataset, though detritivores were important in September (Table S6). In August, however, non-lepidopteran chewing herbivores were the most important determinant of treatment classification, followed by free-feeding caterpillars and predatory insects (Table S6).

**Table 1:** Relative contribution of guilds to differences in community composition between treatments across all three censuses, as measured by random forest mean decrease in accuracy at each node, and by the mean decrease in Gini at each node (Breiman 2001)

Guild	Mean decrease—accuracy	Mean decrease—gini
Spiders	7.18	4.84
Non-lepidopteran chewing herbivores	9.62	5.13
Detritivores	− 0.76	2.58
Free feeding caterpillars	21.90	8.53
Predatory insects	6.30	4.65
Leaf miners	− 2.54	2.81
Non leaf tying shelter builders	− 0.52	3.25
Sucking herbivore	− 4.26	2.70

Higher numbers represent higher importance. See Tables S2 for analyses by months

### Hypothesis 2: leaf quality influences leaf tie formation and colonization

#### Seasonal changes in leaf quality

We found no evidence that the treatments themselves influenced leaf quality, as there were no significant differences (*P* > 0.05) in any of the measured leaf quality variables between treatments (Table S7). There were, however, very strong seasonal changes in leaf quality with leaf quality moderately declining by September depending on the particular factor (Table S6). Repeated measures ANOVA indicated that hydrolysable tannins and total phenolics declined over the course of the season, while condensed tannins accumulated (condensed tannins: *F*_1,137_ = 92.58, *P* < 0.001, 100% increase; hydrolysable tannins: *F*_1,137_ = 148.4, *P* < 0.001, 41% decrease; total phenolics: *F*_1,137_ = 83.39, *P* < 0.001, 44.1% decrease) (Table S6). Percentage dry weight nitrogen (by 73.4%) and carbon (by 1.6%) also declined over the season (%N: *F*_1,137_ = 229.2, *P* < 0.001, %C: *F*_1,137_ = 53.78, *P* < 0.001). Leaf water content slightly increased over the course of the season, from a mean of 58.1% in June to 61.6% in September (*F*_1,137_ = 9.144, *P* = 0.028). Leaf toughness did not significantly change during the season, remaining relatively consistent with a mean value of 188 g/mm^2^.

Parametric correlations suggested that individual trees had consistent within-season hydrolysable tannin and total phenolic concentrations (hydrolysable tannins: r = 0.360, *P* = 0.002, total phenolics: r = 0.264, *P* = 0.028). June and September condensed tannin concentrations were not correlated. Concentrations of condensed tannins were positively correlated with the percentage of dry weight carbon, but only in June (r = 0.469, *P* < 0.001).

In July and September, PCAs of leaf quality variables explained over 75% of the variance using the first two principle components (Table S8). PC1 in both months was significantly negatively correlated with nitrogen levels but positively correlated with hydrolysable tannins and to a lesser extent, condensed tannins (Table S7).

#### Leaf quality, leaf ties, and arthropod composition

Hypothesis 2 was supported by our September but not July observations. Percent tied leaves was not significantly correlated with leaf quality PCs (PC1 *P* = 0.055, PC2 *P* = 0.216) on control trees in July (Table S9). In September, percent tied leaves was negatively correlated with PC1 (Table S8: r = -0.404, *P* = 0.016) (high tannin concentrations and low nitrogen content), and negatively correlated with PC2 (Table S9: r = 0.343, *P* = 0.004) (high carbon:nitrogen ratios and low water content).

Density of all arthropods and that of individual guilds were also more strongly affected by September leaf quality than by July leaf quality. Analysis of total arthropod abundance models showed that the effects of PC2 were mildly significant in July (r = -0.235, *P* = 0.049) (Table S9). In September, PC1, which was associated with high tannin concentrations and low nitrogen content, had significant negative effects on arthropod abundance (r = -0.405, *P* > 0.001). Considering the variable loading of these PCs, the pattern is consistent with the conclusion that trees with lower tannin levels and higher levels of nitrogen and water had more arthropods (Tables S7, S9). Of the non-leaf tying caterpillar guilds, only free-feeding caterpillars were strongly affected by leaf quality, and only in September. Density of this guild was negatively correlated with tannins and positively with nitrogen content (i.e., negatively correlated with PC1).

### Hypothesis 3: habitat diversity hypothesis

**Fig. 4:**
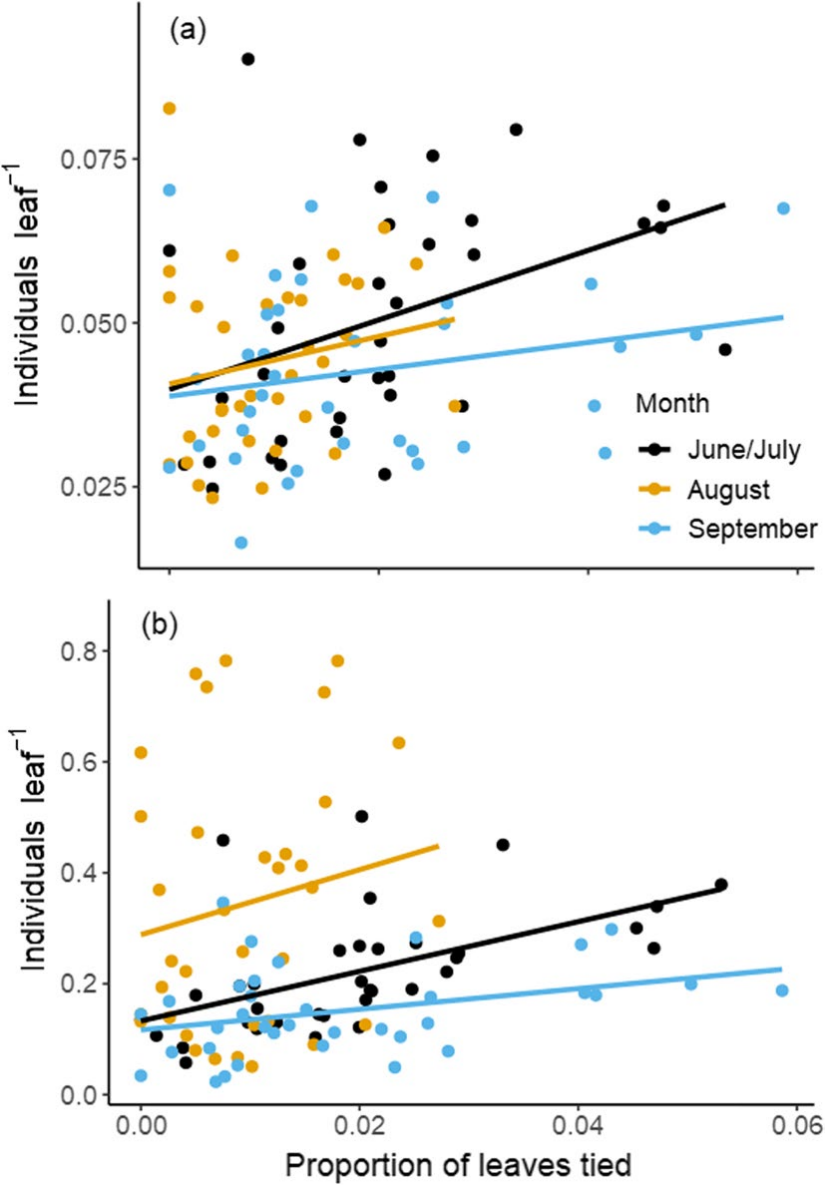
The effect of proportion of leaves tied on each tree, by census month, including leaf tying caterpillars, on **a** the number of individual arthropods (divided by number of leaves on a plant); and **b** species richness (number of species divided by number of leaves on a plant). For a., overall linear model results: proportion of leaves tied *P* = 0.42, month P < 0.001, proportion tied × month *P* = 0.48, R^2^ = 0.237. June/July R^2^ = 0.27, P > 0.001, August R^2^ = 0.001, *P* = 0.31, September R^2^ = 0.09, *P* = 0.04. For b., overall linear model results: proportion of leaves tied *P* = 0.002, month *P* = 0.11, proportion tied × month *P* = 0.45, R^2^ = 0.09. June/July R^2^ = 0.13, *P* = 0.019, August R^2^ = 0.01, *P* = 0.28, September R^2^ = 0.02, *P* = 0.20. See Fig. S3 for contrasting analysis omitting leaf tying caterpillars

We found a positive linear relationship between percent tied leaves and arthropod species richness on control trees for June/July (R^2^ = 0.13, *P* = 0.019), but not for August (R^2^ = 0.01, *P* = 0.28) or September (R^2^ = 0.02, *P* = 0.20) (Fig. 4a). A relatively strong positive relationship was also found between percent leaves tied and total arthropod abundance per leaf on control trees for June/July (R^2^ = 0.27, *P* > 0.001), and a marginal one for September (R^2^ = 0.09, *P* = 0.04) but not August (R^2^ = 0.001, *P* = 0.31) (Fig. 4b). Relationships excluding leaf tying caterpillars from the analyses were similar (Fig. S3). We found no evidence for a curvilinear relationship for any relationship, as the quadratic term did not explain a significant additional amount of variation for any response variable for any time period (*P* > 0.345).

### Hypothesis 4: stress-gradient hypothesis

**Fig. 5:**
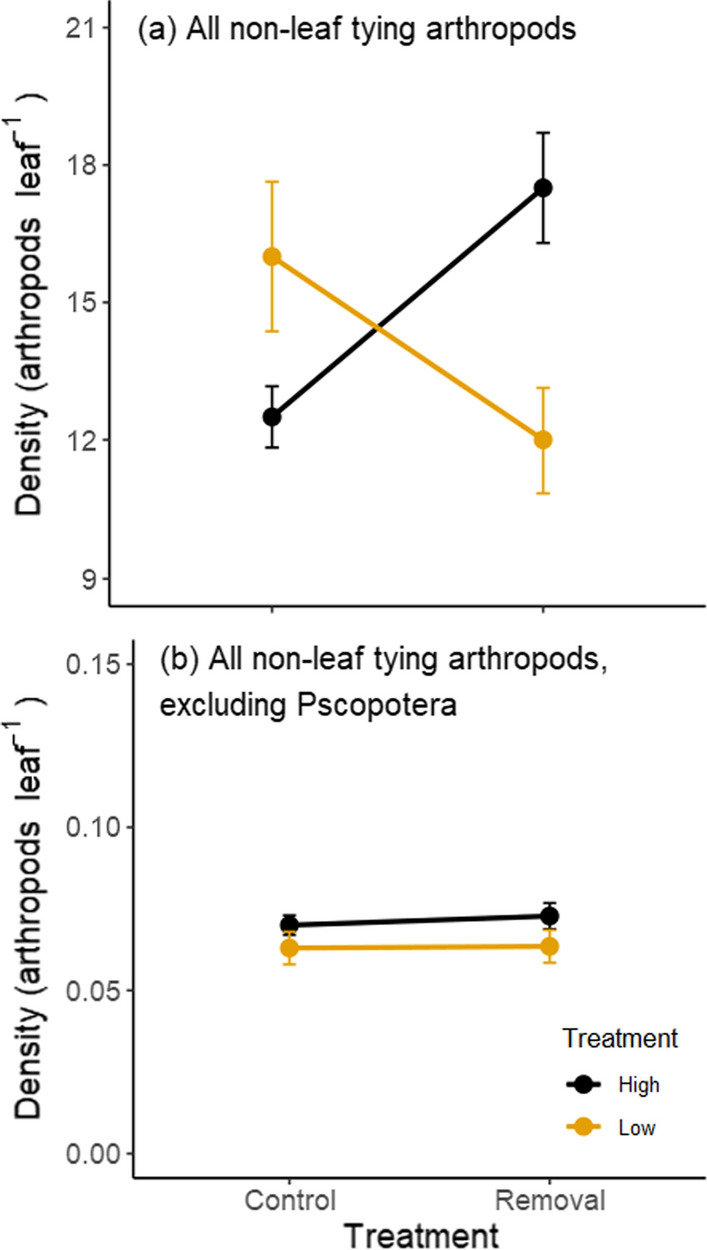
Effect of leaf tie removal on arthropod density (number per leaf of all non-leaf tying arthropods) between control and treatment trees of different quality levels. See Table S3 for quality differences between to the four tree groups. **a** Analysis for all non-leaf tying arthropods, including Psocoptera. Treatment × quality effect was significant at *P* = 0.0110. **b** Analysis for all non-leaf tying arthropods, excluding Pscocoptera. Treatment × quality effect was not significant; *P* = 0.4445

We partitioned control and treatment trees into each of two groups based on leaf characteristics, the low quality group having high concentrations of condensed and hydrolysable tannins, high toughness, and low nitrogen, with the high quality group the opposite for all four variables (Table S3). Two-way repeated measures ANOVAs (leaf quality group versus leaf tie removal) showed significant effects of leaf quality on species richness (*F*_1,66_ = 4.235, *P* = 0.044), but neither treatment (*F*_1,66_ = 0.287, *P* = 0.594) nor treatment × quality interactions (*F*_1,66_ = 0.887, *P* = 0.350) were significant. Treatment (*F*_1,66_ = 9.751, *P* = 0.003) and treatment × quality (*F*_1,66_ = 3.273, *P* = 0.074) had significant and marginally significant impacts, respectively, on species diversity (inverse Simpson’s) across the season, but quality alone had no effects (*F*_1,66_ = 0.072, *P* = 0.789). Here, the effect, although weak based on the interaction term, of removal of leaf ties was greatest for high quality plants, a result opposite to that predicted by the SGH. In contrast, the effects of leaf tie removal on arthropod abundance were more consistent with the SGH. Arthropod abundance was not affected by leaf quality (*F*_1,66_ = 0.400, *P* = 0.528) or treatment (*F*_1,66_ = 0.062, *P* = 0.804) alone, but there was a significant treatment × quality interaction effect (*F*_1,66_ = 6.789, *P* = 0.011) (Fig. 5). When leaf quality was low, control trees had higher abundances of arthropods (0.16 individuals/leaf), but when leaf quality was high, removal trees had higher abundances (0.18 individuals/leaf) (Fig. 5a). Most of these interaction effects were apparently due to Pscoptera (compare Fig. 5a with Fig. 5b).

## Discussion

In the current study, experimental removal of leaf tying caterpillars on *Quercus alba* in Missouri, USA, revealed that the presence of their constructs significantly increased diversity and modified structure of the associated arthropod communities at the whole plant level. Most importantly, the observed effects of ecosystem engineering were influenced by the leaf quality of the host plants, and were so in four ways. First, as a starting point, colonization of control trees by leaf tying caterpillars was negatively correlated with leaf quality in September. Second, the colonization or avoidance of leaf ties by non-leaf tying arthropods was partially dependent on leaf quality, particularly for free-feeding caterpillars in September. Third, both species richness and arthropod abundance at the whole tree level increased with increasing numbers of leaf ties, as influenced by leaf quality, consistent with the Habitat Diversity Hypothesis (Williams 1964; Dianzinga et al. 2020). However, the percentage of tied leaves was low throughout the experiment so the effects of a large percentage of leaves in ties on the community remain unknown. Fourth, low quality control trees (those with leaf ties) had a higher abundance of non-leaf tying arthropods than low quality removal trees. In contrast, high quality control trees had lower abundance of secondary inhabitants than high quality tie removal trees. These latter results are consistent with the Stress-Gradient Hypothesis, i.e., that the effects of ecosystem engineering are greater in more stressful environments. Overall, these results suggest that the positive impact of leaf tying caterpillars on *Q. alba* is modified by host plant quality.

### Arthropod communities

In a previous study (Lill and Marquis 2003), removal of leaf tying caterpillars for a three-week period from *Quercus alba* saplings early in the season reduced species richness of herbivores for the duration of the season. Here, we found less dramatic effects of leaf tie removal on species richness and diversity across the 106 species we encountered on our study plants, perhaps because of the lower number of leaf ties occurring naturally in the current study compared to the previous one. There were no effects on species richness for any census, but both Shannon diversity and inverse Simpson diversity, metrics that take into account species richness as well as the relative abundance of species, were reduced in the September census. Thus, at the whole community level, removal of leaf ties decreased species diversity but only at the end of the season, suggesting a temporally cumulative effect of leaf tie removal during the growing season.

Removal of leaf ties had a major impact on community structure, affecting almost all components of that community. Five of eight guilds were significantly affected by leaf tie removal, and for seven of eight of these guilds, the effect of this treatment was modified by season. While the overall effect of tie removal was negative on abundance across all guilds (in June-July), the response of individual guilds was unique. Abundances of four guilds decreased with leaf tie removal while abundances of three other guilds increased.

The results suggest that the treatment effect is more than a shifting of arthropods within trees, but is one of differential colonization of trees, and that colonization preference varies by guild. Given the phenology of arthropod activity (many species are ovipositing in June and afterwards; Marquis et al. 2019b) and the relatively long course of the experiment (four months), we believe the study was sufficiently long to affect colonization at the whole tree level. All else being equal, particularly leaf quality, some species (non-leaf tying shelter builders, predatory insects, sucking herbivores, and detritivores) would be attracted to trees with more leaf ties, while others (spiders, leaf miners, and free-feeding caterpillars) would avoid trees with a large number of leaf ties. A previous study in this system (Lill and Marquis 2004) showed that composition of tied and non-tied leaves of white oak is in part due to actual oviposition in ties.

### Leaf ties and the habitat diversity hypothesis

The proportion of leaves tied on control trees was equally high in July and September, but dipped in August (see also Lill and Marquis 2003; Lill 2004). The August census occurred after the first generation of leaf tying caterpillars and before the emergence of the second generation of leaf tying caterpillars. The number of ties dips between the two generations because no new ties are being formed and a portion of the previously formed ties naturally come apart.

When leaf tying caterpillars were most active, species richness (June/July and September) and arthropod abundance (June/July) shared a positive relationship with the percentage of leaves tied. These relationships were not found in August, perhaps due to the fact that leaf tying caterpillars are between generations at that time, lowering the number of tied leaves. The habitat diversity hypothesis predicts diversity will peak at equivalent levels of two or more habitat types, which has been supported by a number of studies of ecosystem engineers (e.g., Wright et al. 2002, 2003; Castilla et al. 2004; review by Romero et al. 2015). The fact that species richness and abundance generally increased with an increasing proportion of tied leaves in this study is supportive. However, we found no evidence for a curvilinear relationship. Because the natural amount of leaves tied on a tree rarely exceeded 5% (only 1 tree in July, 0 in August, and 2 in September exceeded 5% of leaves tied), we suggest that an intermediate point was not reached.

### Leaf quality, leaf ties, and arthropod communities and the stress-gradient hypothesis

A number of studies have shown that the impact of ecosystem engineering by both vertebrates and invertebrates is greater in more stressful environments (e.g., Vieira and Romero 2013; Arribas et al. 2014; Wright and Gribben 2017; McAfee et al. 2019; Lowney and Thompson 2022; but see Albertson et al. 2021). Vieira and Romero (2013) found greater effects of leaf rolls on associated arthropod diversity in the dry season (harsher compared to the wet season) in what appears to be the only previous test of the Stress-Gradient Hypothesis involving insects as ecosystem engineers. Here we chose leaf quality to represent a stress gradient. When the arthropod community was compared between low and high quality plants in addition to treatment, a difference in the ecosystem engineering effect was found based on changes in arthropod abundance (we found weak or no effects based on species richness and inverse Simpson diversity). Arthropod abundance was higher on control trees when leaf quality was low, but higher on removal trees when quality was high. These differences in mean arthropod abundance between quality levels were due to high abundances of Psocoptera during the study. We do not know enough about the biology of Psocoptera in our system, which consist of two morphospecies, to explain this result, other than that they do seek out leaf ties, and often build their own silk shelters within them. Overall, the differences in abundance between treatment and quality levels suggest that environmental context (i.e., foliage quality) can change the dynamics of how ecosystem engineering affects ecological communities. A similar result has been found for the engineering effects of plants on bivalves: in sub-tidal habitats, positive interactions increased with increasing temperature but decreased with increasing temperature in intertidal habitats (Gagnon et al. 2020).

## Conclusions

This study demonstrates that ecosystem engineering can affect the diversity and community structure of *Quercus alba* arthropod communities at the whole-plant level, and that these effects were influenced by the quality of foliage on study trees. While previous work in this system has shown that removal of leaf tying caterpillars for three weeks early in the season can have significant impacts on arthropod communities (Lill and Marquis 2003), we show here that arthropod communities on plants with and without leaf tying caterpillars differed throughout the season; by September, trees with leaf ties had arthropod communities that were significantly more diverse, and the species assemblages between treatments shared relatively little compositional similarity. Furthermore, this is the first study to show that the quality of plant foliage can impact the way that ecosystem engineering affects arthropod communities, in addition to the direct influence of leaf quality on arthropods documented in previous studies (Forkner et al. 2004). The results of this study also demonstrate how indirect interactions between herbivore species can interact with bottom-up trophic forces, resulting in arthropod communities that can be significantly different from one another depending on the degree of ecosystem engineering by leaf tying caterpillars and host plant quality. Future experiments should artificially increase the number of leaf ties on plants to higher levels to determine the impact on community structure as a function of the proportion of the landscape that is engineered.

It seems likely that other environmental factors could also have an impact on ecosystem engineering and arthropod community dynamics. Future studies should consider how abiotic factors (e.g., canopy vs. understory, dry vs. wet environments, open vs. closed canopy, nutrient rich vs. nutrient poor soils), as they represent stressors, influence the ecosystem engineering impact of shelter building insects on trees. In addition to leaf quality, plant architecture affects colonization by leaf tying caterpillars on *Quercus alba* (Marquis and Lill 2010) and may have an indirect impact on arthropod community diversity. Future studies in this system should include leaf and branching architecture in conjunction with leaf quality, and attempt to identify other factors that may influence the effects of ecosystem engineering.

Our results suggest an important link between ecosystem engineering on plants and the tritrophic ecology of the associated organisms (Marquis and Lill 2007, pathway 4 of Fig. 1.1; Sanders et al. 2014). We found that predator abundance at the plant level was affected by the presence of leaf ties. This leads us to predict that the impact of the third trophic level on the structure of the arthropod community will change in the presence of ties. In our system, whether predation pressure increases or decreases is not immediately predictable, given that spiders decreased in the presence of leaf ties while predatory insects increased. Future studies should assess the effects of natural enemies on the composition of arthropod communities in and outside of leaf shelters of control trees (see Tvardikova and Novotny 2012), and on experimental trees from which leaf constructs have been removed.

### Supplementary Information

Below is the link to the electronic supplementary material.

Supplementary file1 (DOCX 229 KB)

Supplementary file2 (DOCX 66 KB)

Supplementary file3 (XLSX 15 KB)

Supplementary file4 (DOCX 43 KB)

## Data Availability

Data are available upon request from the authors.
