# Supplementary material for: Ecosystem engineering and leaf quality together affect arthropod community structure and diversity on white oak (Quercus alba L.)
Source: Oecologia. 2023 Sep 9;203(1-2):13–25. doi: 10.1007/s00442-023-05439-1 (PMC10615914; doi:10.1007/s00442-023-05439-1)
Supplement: Supplementary file 1 — Supplementary file1 (DOCX 229 KB) [file 442_2023_5439_MOESM1_ESM.docx]

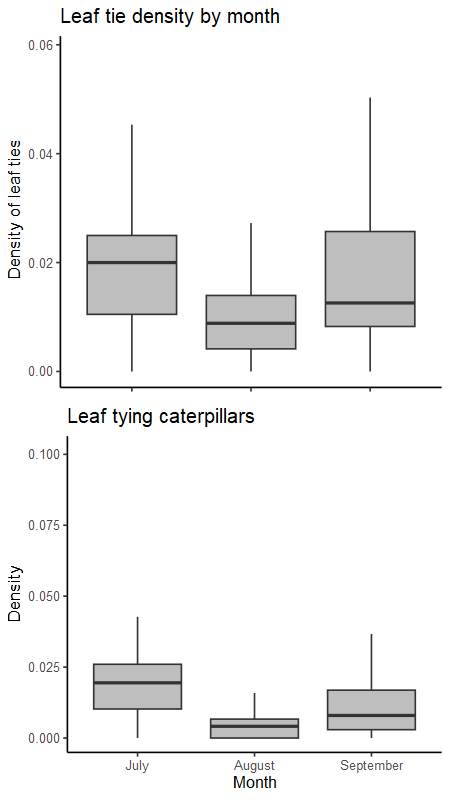


(a)

(b)

Figure S1. Seasonal distribution of leaf tying caterpillar activity for control trees. These are boxplots, showing medians (horizontal line) within the box. The upper edge of the box represents 75% of the data and lower edge 25% of the data, with the two horizontal lines containing 95% of the data. (a) Density (number per leaf) of leaf ties by census (census effect: *P* < 0.001) (Table S4). (b) Density of leaf tying caterpillars by census (census effect: *P* < 0.05) (Table S4).


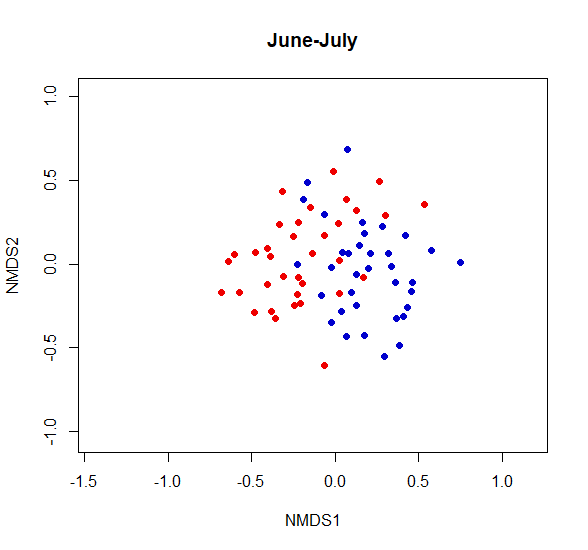


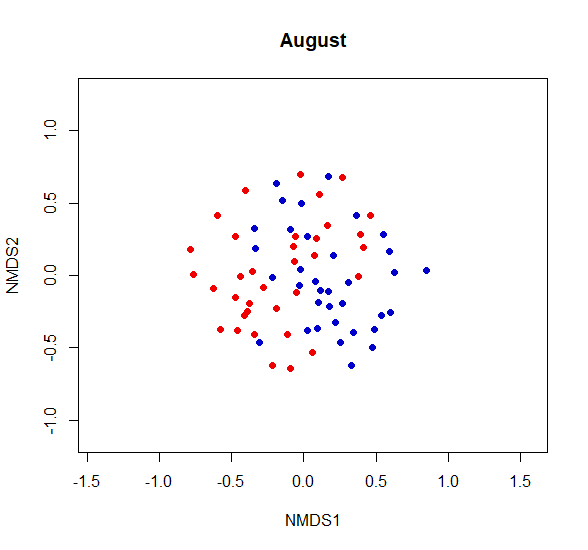


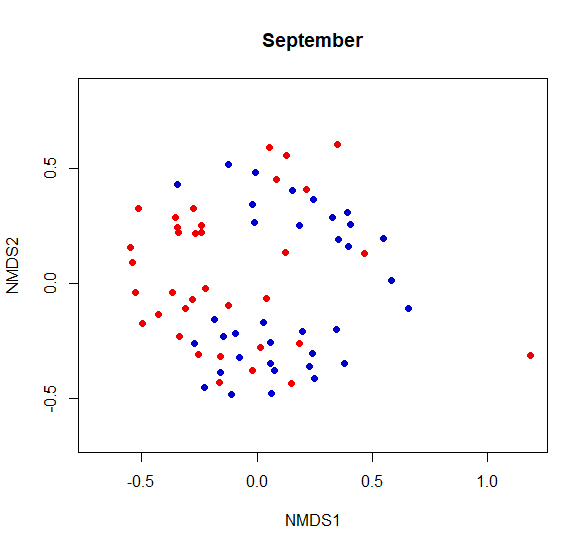


Figure S2. Nonmetric multidimensional scaling plot for arthropod community data encompassing for a) June-July, b) August, and c) September. Blue dots represent control trees, red dots represent treatment trees.


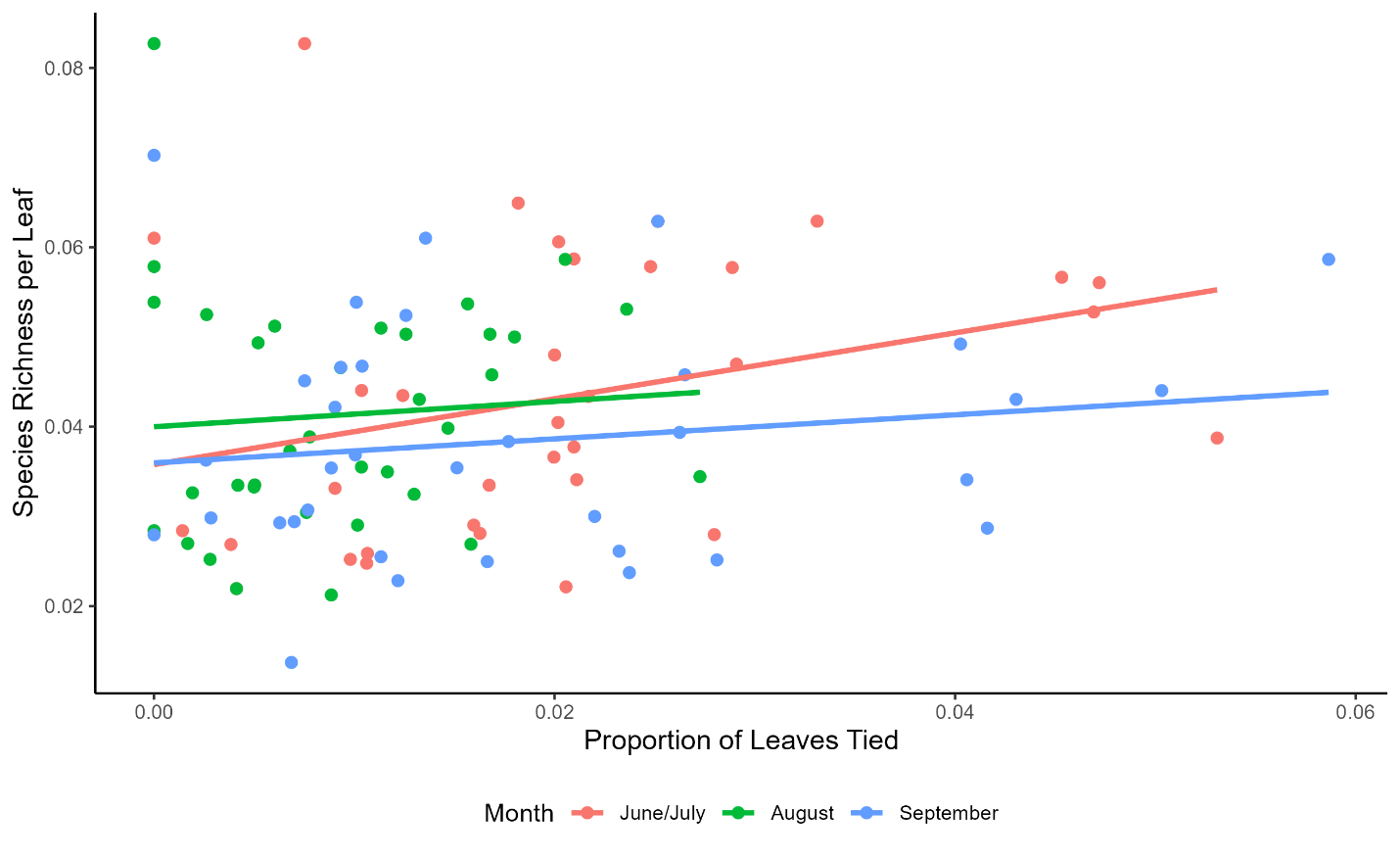

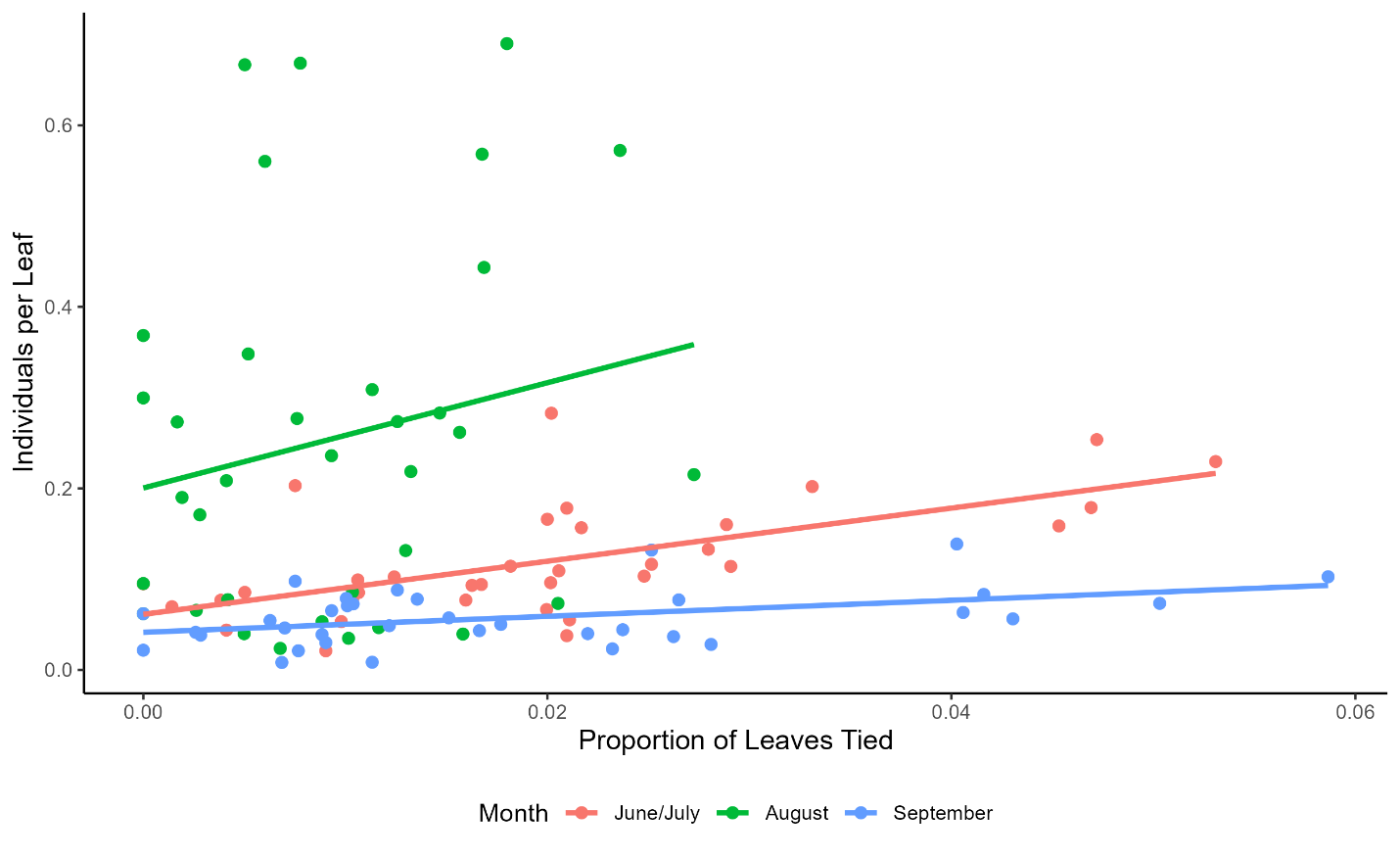


Figure S3. The effect of proportion of leaves tied on each tree, by census month, excluding leaf tying caterpillars from the analysis, on a. the number of individual arthropods (divided by number of leaves on a plant); and b. species richness (number of species divided by number of leaves on a plant). For a., overall linear model results: proportion of leaves tied *P* = 0.86, month *P* < 0.001, proportion tied × month *P* = 0.28, R² = 0.333. June/July R² = 0.36, *P* > 0.001, August R² = 0.01, *P* = 0.25, September R² = 0.16, *P* = 0.01. For b., overall linear model results: proportion of leaves tied *P* = 0.063, month *P* = 0.28, proportion tied × month *P* = 0.60, R² = 0.021. June/July R² = 0.071, *P* = 0.067, August R² = 0.01, *P* = 0.66, September R² = 0.01, *P* = 0.38.
