# Supplementary material for: Ecosystem engineering and leaf quality together affect arthropod community structure and diversity on white oak (Quercus alba L.)
Source: Oecologia. 2023 Sep 9;203(1-2):13–25. doi: 10.1007/s00442-023-05439-1 (PMC10615914; doi:10.1007/s00442-023-05439-1)
Supplement: Supplementary file 2 — Supplementary file2 (DOCX 66 KB) [file 442_2023_5439_MOESM2_ESM.docx]

Table S1. Number of lepidopteran larvae removed per species at each of the six removal dates from the

35 Removal plants, plus grand totals.

| Date | 6 17 09 | 6 24 09 | 7 17 09 | 7 22 09 | 8 28 09 | 9 6 09 |  |
| --- | --- | --- | --- | --- | --- | --- | --- |
| Species | Number removed | Number removed | Number removed | Number removed | Number removed | Number removed | Grand total |
| *Arogalea cristifasciella* | 7 | 6 | 3 | 5 | 0 | 1 | 22 |
| *Pseudotelphusa quercinigracella* | 4 | 56 | 9 | 3 | 3 | 6 | 81 |
| *Psilocorsis reflexella/cryptolechiella* | 7 | 18 | 5 | 6 | 5 | 8 | 49 |
| *Psilocorsis quercicella* | 4 | 1 | 2 | 4 | 1 | 3 | 15 |
| *Erynnis juvenalis* | 1 | 0 | 0 | 0 | 0 | 0 | 1 |
| *Antaeotricha osseella* | 0 | 0 | 2 | 0 | 0 | 6 | 8 |
| Unknown | 58 | 2 | 0 | 0 | 1 | 9 | 70 |
| Total | 81 | 84 | 21 | 18 | 11 | 33 | 248 |
