# Supplementary material for: Ecosystem engineering and leaf quality together affect arthropod community structure and diversity on white oak (Quercus alba L.)
Source: Oecologia. 2023 Sep 9;203(1-2):13–25. doi: 10.1007/s00442-023-05439-1 (PMC10615914; doi:10.1007/s00442-023-05439-1)
Supplement: Supplementary file 4 — Supplementary file4 (DOCX 43 KB) [file 442_2023_5439_MOESM4_ESM.docx]

Table S3. Correlations (Pearson correlation coefficients) among leaf quality factors. The bottom/left half of the matrix is the inverse of the top/right half. N = nitrogen, C = carbon.

July

|  | Hydrolysable tannins | Condensed tannins | Total phenolics | N:hydrolysable tannins | C:N | Nitrogen | Water | Toughness |
| --- | --- | --- | --- | --- | --- | --- | --- | --- |
| Hydrolysable tannins | x | 0.334** | 0.653*** | -0.896*** | 0.091 | -0.068 | -0.138 | 0.056 |
| Condensed tannins |  | x | 0.331** | -0.272* | -0.044 | 0.106 | 0.046 | 0.178 |
| Total phenolics |  |  | x | -0.634*** | 0.232 | -0.180 | -0.143 | -0.169 |
| N:hydrolysable tannins |  |  |  | x | -0.406*** | 0.420*** | 0.070 | 0.280 |
| C:N |  |  |  |  | x | -0.988*** | -0.168 | 0.081 |
| Nitrogen |  |  |  |  |  | x | 0.172 | 0.209 |
| Water |  |  |  |  |  |  | x | 0.563* |
| Toughness |  |  |  |  |  |  |  | x |

***: P < 0.001, **: P < 0.01, *: P < 0.05

September

|  | Hydrolysable tannins | Condensed tannins | Total phenolics | N:hydrolysable tannins | C:N | Nitrogen | Water | Toughness |
| --- | --- | --- | --- | --- | --- | --- | --- | --- |
| Hydrolysable tannins | x | 0.235* | 0.511*** | -0.907*** | 0.099 | -0.088 | -0.129 | -0.034 |
| Condensed tannins |  | x | 0.262* | -0.320** | 0.186 | -0.221 | -0.092 | 0.031 |
| Total phenolics |  |  | x | -0.523*** | 0.221 | -0.219 | 0.164 | 0.046 |
| N:hydrolysable tannins |  |  |  | x | -0.467*** | 0.456*** | 0.175 | 0.007 |
| C:N |  |  |  |  | x | -0.978*** | 0.032 | 0.084 |
| Nitrogen |  |  |  |  |  | x | -0.057 | -0.141 |
| Water |  |  |  |  |  |  | x | 0.506* |
| Toughness |  |  |  |  |  |  |  | x |

***: P < 0.001, **: P < 0.01, *: P < 0.05

Table S4. Univariate ANOVA results (F and *P* values) testing for differences between quality groups in Control and Removal treatments, used to test Hypothesis 4.

| July | | | | | |
| --- | --- | --- | --- | --- | --- |
|  | Control | |  | Removal | |
| Variable | F1,33 | P |  | F1,33 | P |
| Condensed tannins | 1.032 | 0.317 |  | 3.02 | 0.092 |
| Hydrolysable tannins | 15.103 | **< 0.001** |  | 10.383 | **0.003** |
| Total phenolics | 12.119 | **0.001** |  | 4.84 | **0.035** |
| Nitrogen content | 17.505 | **< 0.001** |  | 14.225 | **< 0.001** |
| Carbon content | 3.039 | 0.091 |  | 3.612 | 0.066 |
| Water content | 4.217 | **0.048** |  | 3.157 | 0.085 |
| Toughness | 5.86 | **0.021** |  | 4.877 | **0.034** |
|  |  |  |  |  |  |
| September | | | | | |
|  | Control | |  | Removal | |
| Variable | F1,33 | P |  | F1,33 | P |
| Condensed tannins | 15.122 | **< 0.001** |  | 19.601 | **< 0.001** |
| Hydrolysable tannins | 6.795 | **0.014** |  | 13.307 | **< 0.001** |
| Total phenolics | 4.26 | **0.047** |  | 6.759 | **0.014** |
| Nitrogen content | 21.518 | **< 0.001** |  | 12.239 | **0.001** |
| Carbon content | 0.221 | 0.642 |  | 0.013 | 0.91 |
| Water content | 0.948 | 0.337 |  | 0.619 | 0.437 |
| Toughness | 3.99 | 0.055 |  | 3.528 | 0.069 |

Table S5. Results (F values and P values, indicated by asterisks) of analysis of variance (ANOVA) for the effect of Treatment (control vs. removal), Season (June-July, August, and September), and the interaction between Treatment and Season on density (numbers per leaf) of all guilds of arthropods found on control and removal trees. Only season effects for the density of leaf ties and leaf tying caterpillars are shown because both were experimentally removed from ‘removal’ trees. N = 70 for treatment effects and N = 3 for season effects.

|  | Density | | | | | | | | | | | |
| --- | --- | --- | --- | --- | --- | --- | --- | --- | --- | --- | --- | --- |
| ANOVA factor | Leaf ties | Total arthropods | Leaf tying  caterpillars | Non-leaf  tying shelter  builders | Free-feeding  caterpillars | Free-feeding  chewing non-Lepidoptera | Leaf miners | Sucking  herbivores | Spiders | Non-spider predators | Detritivores |  |
| Treatment | -- | 0.407 | -- | 0.48 | 7.533** | 0.04 | 0.021 | 8.098** | 8.12** | 37.21*** | 5.386* |  |
| Season | 7.69*** | 39.18*** | 3.46* | 6.869** | 12.52*** | 65.45*** | 15.68*** | 0.783 | 0.441 | 17.29*** | 42.2*** |  |
| Treatment × season | -- | 15.98*** | -- | 3.44** | 8.376*** | 26.0*** | 6.428*** | 2.568* | 1.96 | 21.38*** | 20.9*** |  |

***: P < 0.001, **: P < 0.01, *: P < 0.05

Table S6. Separate analysis by month for community composition importance as measured by random forest mean decrease in accuracy at each node, and by the mean decrease in Gini at each node (Breiman 2001). Higher numbers represent higher importance.

A. June/July

| Guild | Mean decrease – accuracy | Mean Decrease – Gini |
| --- | --- | --- |
| Spiders | 5.65 | 4.97 |
| Chewing non-lepidopteran herbivores | 4.33 | 4.69 |
| Detritivores | -3.80 | 3.35 |
| Free-feeding caterpillars | 17.25 | 8.52 |
| Predatory insects | -0.27 | 4.70 |
| Leaf miners | -5.57 | 2.07 |
| Non leaf tying shelter builders | 0.03 | 3.43 |
| Sucking herbivores | -6.18 | 2.77 |

B. August

| Guild | Mean decrease – accuracy | Mean decrease – Gini |
| --- | --- | --- |
| Spiders | 4.62 | 3.65 |
| Chewing non-lepidopteran herbivores | 19.82 | 8.72 |
| Detritivores | -6.22 | 2.92 |
| Free-feeding caterpillars | 8.62 | 4.70 |
| Predatory insects | 8.38 | 5.51 |
| Leaf miners | -4.75 | 2.20 |
| Non leaf tying shelter builders | -0.47 | 3.21 |
| Sucking herbivores | -0.79 | 3.59 |

C. September

| Guild | Mean decrease – accuracy | Mean decrease – Gini |
| --- | --- | --- |
| Spiders | 9.29 | 4.97 |
| Chewing non-lepidopteran herbivores | 7.44 | 4.60 |
| Detritivores | 6.47 | 3.04 |
| Free-feeding caterpillars | 30.51 | 9.60 |
| Predatory insects | 8.83 | 5.99 |
| Leaf miners | -4.06 | 1.14 |
| Non leaf tying shelter builders | 0.90 | 1.80 |
| Sucking herbivores | 1.74 | 3.31 |

Table S7. Treatment and season effects (F values, asterisks indicate P values) on the measured leaf quality factors, and correlations (Pearson correlation coefficients) of each quality factor with principal components 1 and 2 (PCI and PC2, respectively), based on principal component analysis run on leaf samples collected in July, and then again in September. N = nitrogen, C = carbon.

|  | Leaf quality factor | | | | | | | |
| --- | --- | --- | --- | --- | --- | --- | --- | --- |
| ANOVA factor or correlation | Hydrolysable tannins | Condensed tannins | Total phenolics | N:Hydrolysable tannins | C:N | Nitrogen | Water | Toughness |
| Treatment (F) | 0.032 | 0.086 | 0.913 | 0.06 | 0.251 | 0.245 | 0.170 | 0.011 |
| Season (F) | 148.4*** | 92.58*** | 83.39*** | 26.88*** | 164.9*** | 229.2*** | 9.144** | 3.541 |
| Treatment × season (F) | 0.416 | 0.159 | 0.256 | 0.198 | 0.133 | 0.242 | 0.255 | 1.250 |
| PC1 correlation (July) | 0.622*** | 0.297* | 0.551*** | -0.856*** | 0.766*** | -0.744*** | -0.440*** | 0.337** |
| PC2 correlation (July) | -0.340** | -0.863*** | -0.252* | 0.199 | 0.275* | -0.324** | -0.493*** | 0.136 |
| PC1 correlation (Sept) | 0.605*** | 0.652*** | 0.449*** | -0.815*** | 0.716*** | -0.724*** | -0.150 | 0.283 |
| PC2 correlation (Sept) | 0.089 | 0.219 | -0.191 | 0.052 | -0.334** | 0.344** | -0.931*** | 0.157 |

***: P < 0.001, **: P < 0.01, *: P < 0.05

Table S8. Leaf quality variable loadings in July and September for the first and second principal components (PCs), together with the proportion of variance explained by each component.

|  | July | |  | September | |
| --- | --- | --- | --- | --- | --- |
| Variable | PC1 | PC2 |  | PC1 | PC2 |
| N:hydrolysable tannins | -0.677 | 0.190 |  | -0.639 | 0.051 |
| Condensed tannins | 0.235 | -0.822 |  | 0.511 | 0.215 |
| Carbon:nitrogen | 0.605 | 0.261 |  | 0.562 | -0.330 |
| Water | -0.348 | -0.470 |  | -0.117 | -0.918 |
| Proportion of variance | 0.400 | 0.38 |  | 0.410 | 0.362 |

Table S9. Correlations (Pearson correlation coefficients) between principal components (PC1 and PC2) from principal component analysis with leaf ties density, and densities of total arthropods, and of each guild. Correlations are calculated for June/July and September arthropod densities only because leaf quality factors were measured in July and September only. Note that analyses for leaf ties and leaf tying caterpillars were conducted for control trees only.

|  | June-July density | | | | | | | | | | |
| --- | --- | --- | --- | --- | --- | --- | --- | --- | --- | --- | --- |
|  | Leaf ties | Total arthropod  abundance | Leaf tying  caterpillars | Non-leaf  tying shelter  builders | Free-feeding  caterpillars | Free-feeding  chewing non-Lepidoptera | Leaf miners | Sucking  herbivores | Spiders | Non-spider predators | Detritivores |
| PC1 | -0.055 | 0.038 | -0.030 | 0.146 | 0.055 | 0.066 | 0.140 | 0.257* | -0.104 | -0.006 | 0.200 |
| PC2 | -0.216 | -0.235* | 0.031 | -0.064 | -0.149 | -0.252* | -0.216 | -0.083 | -0.080 | -0.162 | 0.077 |
|  | September density | | | | | | | | | | |
|  | Leaf ties | Total arthropod  abundance | Leaf tying  caterpillars | Non-leaf  tying shelter  builders | Free-feeding  caterpillars | Free-feeding  chewing non-Lepidoptera | Leaf miners | Sucking  herbivores | Spiders | Non-spider predators | Detritivores |
| PC1 | -0.404* | -0.405*** | 0.092 | 0.034 | -0.401*** | 0.233 | -0.255* | -0.129 | -0.017 | -0.113 | -0.263* |
| PC2 | 0.178 | 0.094 | 0.343** | -0.121 | 0.117 | -0.211 | 0.076 | 0.235* | 0.090 | 0.100 | 0.077 |

***: P < 0.001, **: P < 0.01, *: P < 0.05
